# Possible role of selenium in ameliorating lead-induced neurotoxicity in the cerebrum of adult male rats: an experimental study

**DOI:** 10.1038/s41598-023-42319-3

**Published:** 2023-09-21

**Authors:** Abdelmonem Awad Hegazy, Ayat M. Domouky, Fatma Akmal, Dalia Ibrahim El-wafaey

**Affiliations:** 1 Human Anatomy and Embryology Department, Faculty of Dentistry, Zarqa University, Zarqa City, 13110 Jordan; 2 Human Anatomy and Embryology Department, Faculty of Medicine, Zagazig University, Zagazig City, 44519 Egypt

**Keywords:** Cell biology, Structural biology

## Abstract

Chronic lead (Pb) poisoning is one of the greatest public health risks. The nervous system is the primary and most vulnerable target of Pb poisoning. Selenium (Se) has been shown to be a potential protection against heavy metal toxicity through anti-inflammatory and antioxidant properties. Therefore, the present study aimed to elucidate the possible protective role of Se in ameliorating the effects of Pb on rat cerebral structure by examining oxidative stress and markers of apoptosis. The rats were divided into 6 groups: control group, Se group, low Pb group, high Pb group, low Pb + Se group, high Pb + Se group. After the 4-week experiment period, cerebral samples were examined using biochemical and histological techniques. Pb ingestion especially when administered in high doses resulted in cerebral injury manifested by a significant increase in glial fibrillary acidic protein, malondialdehyde (MDA) marker of brain oxidation and DNA fragmentation. Moreover, Pb produced alteration of the normal cerebral structure and cellular degeneration with a significant reduction in the total number of neurons and thickness of the frontal cortex with separation of meninges from the cerebral surface. There was also a decrease in total antioxidant capacity. All these changes are greatly improved by adding Se especially in the low Pb + Se group. The cerebral structure showed a relatively normal histological appearance with normally attached pia and an improvement in neuronal structure. There was also a decrease in MDA and DNA fragmentation and an increase TAC. Selenium is suggested to reduce Pb-induced neurotoxicity due to its modulation of oxidative stress and apoptosis.

## Introduction

Lead (Pb) has been documented by the World Health Organization as one of the most toxic substances to human health^1^. Chronic Pb poisoning is a major public health hazard, especially in developing countries^2^. Pb is a cumulative toxicant that has a wide range of effects on body systems, including the nervous, digestive, cardiovascular, and renal systems^3^. The general population's exposure to Pb comes mostly through water and food^4^. Other important sources of Pb exposure include playing with guns, welding, painting, crafting ammunition, glass polishing, jewelry production, ceramic production, water pipes, batteries, radiation shielding, and stained-glass design. Herbal remedies may also be a cause of major exposure^5^. Pb revealed higher concentration values in agricultural activities, such as the use of many pesticides and fertilizers, as it is one of the greatest minerals absorbed by plant roots^6^. Compared to other body systems, the nervous system appears to be the main and most sensitive target of Pb poisoning^7^. Pb crosses the blood–brain barrier (BBB) and replaces calcium and zinc ions causing neurological deficits^8^. Furthermore, Pb has the potential to cause apoptosis^9^. Imbalances from changes in antioxidant homeostasis, resulting from Pb toxicity, can lead to increased free radical generation, lipid peroxidation, and oxidative stress. The brain is highly sensitive to the effects of oxidative stress due to its high rates of oxygen consumption, the presence of high levels of unsaturated fatty acids as substrates for lipid peroxidation, and the non-regenerative nature of neurons^10^.

Selenium (Se) is a microelement essential for the homeostasis of many vital endocrine functions, such as the thyroid gland, and signal transduction pathways^11^. It is a nutritionally essential trace metalloid^12^ that has antioxidant and anti-inflammatory activity^13^. It is incorporated into several selenoproteins that play a pivotal role in several biological systems involved in neurodevelopment and cellular redox status^14^. The main source of Se in human is diet like cereals, meat and seafood^12^. It has been suggested that Se plays an important role as an antiaging, antioxidant, protecting liver, repairing cellular damage, and enhancing immune function^15^. Moreover, beneficial clinical results have been reported for the use of Se in the treatment of neurodegenerative disorders such as dementia caused by Alzheimer's disease^14^. Adults who have had seizures have a significantly lower level of Se in their blood. Selenoprotein P, encoded by SEPP1 gene, plays a major role in neuroprotection by inhibiting apoptosis and prolonging neuronal survival. Human research showed that Se status is associated with the risk of dementia and Alzheimer's disease^16^. Se has been shown to reduce the production of reactive oxygen species (ROS), protect cells from glutamate toxicity, oxidative stress, and inflammatory cytokines, and ultimately prevent cell death brought on by these conditions^17^. Moreover, Se has been shown to protect against methylmercury neurotoxicity in experimental studies^18^. However, to our knowledge, previous experimental studies were not sufficient to support its protective effect in case of Pb neurotoxicity. Therefore, the aim of this study was to evaluate the potential deleterious effect of Pb on the cerebral structure of rats. Moreover, we attempted to elucidate any protective role of Se in ameliorating such disorder if it exists by investigating its potential effects on oxidative stress and apoptotic markers through biochemical, immunohistochemical and histological methods.

## Material and methods

### Chemicals

Pb acetate 99% was acquired from Piochem Company. Se Sodium Selenite, white to off-white water-soluble powder with purity more than 98%, purchased from Sigma-Aldrich, USA. Distilled water was brought from Kemecta Company.

### Animals

This experiment involved 60 adult male albino rats, each weighing 150–250 g. They were obtained from animal house of faculty of medicine. All the animals were kept in sanitary conditions. Standard food and drink were provided ad libitum. They were given 15 days to acclimate to the laboratory settings before they were tested. The Institutional Animal Care and Use Committee^19^ approved the trial design with approval number ZU-IACUC/3/F/93/2020. All animals were cared for in accordance with the National Institutes of Health (NIH) Animal Care Guidelines. The study was also conducted according to ARRIVE guidelines^20^.

### Experiment protocol

The rats were randomly divided into 6 groups of 10 animals each: control group, Se group, low Pb group, high Pb group, low Pb + Se group and high Pb + Se group. The post-acclimation period for the experiment was 4 weeks. Control group received balanced diet only without any treatment. Se group was given 0.25 mg/kg/day of Se by oral gavage^14^. Low Pb group animals were given 20 ml/kg/day of Pb acetate dissolved in 1ml distilled water by oral gavage^8^. High Pb group was given 50 ml/kg/day of Pb acetate dissolved in 1ml distilled water by oral gavage^21^, low Pb + Se group was given 20 mg/kg/day of Pb acetate along with 0.25 mg/kg/day of Se by oral gavage^8,14^. High Pb + Se group was given with 50 mg/kg/day of Pb acetate along with 0.25 mg/kg/day of Se by oral gavage^14,21^.

### Animal weight

For measuring body weight, each animal was put in a closed plastic container and weighed day before the experiment (bwt) and at the last day (Lwt). The results were written in a record for each labeled rat. Moreover, the whole brains were harvested after animal slaughtering and weighted before dissection. The relative brain weight was obtained as a percentage of brain weight (br) to Lwt (br/Lwt %)^22^.

### Samples preparation

All animals were anaesthetized by intra-peritoneal injection of thiopental 30 mg kg. Then, the brain was carefully excised and weighted; and the two cerebral hemispheres were split. One-half was wrapped in aluminum foil and frozen at − 80 °C until it was needed for biochemical research. For histopathological examinations, the other was fixed in 10% formol saline for 48 h, or buffered glutaraldehyde solution at pH 7.4 for 2–24 h in a refrigerator at 4 °C^19^.

### Comet assay for DNA fragmentation

By measuring the length of DNA migration and the percentage of migrated DNA, the quantitative and qualitative amount of DNA damage in preprocessed cells was determined. CCD camera was used in conjunction with Comet 5 image analysis software created by kinetic imaging, Ltd. (Liverpool, UK). The research was carried out at the Cairo-based Animal Reproduction Research Institute (ARRI). It was performed to evaluate the apoptotic effect of Pb. Its steps were followed according to guidelines of Tice et al.^23^.

### Homogenate tissue analysis

Tissues were homogenized in ice-cold phosphate buffers (50 mM, pH 7.4) 5 times their tissue weight, and centrifuged at 5000 rpm for 30 min. Then, supernatants were preserved in a deep freeze until being used for the following assays^24^.

#### Lipid peroxidation assay

Thiobarbituric acid (TBA) (0.2%) in H_2_SO_4_ (0.05 M) was combined with cerebral homogenate samples and heated in a boiling water bath for 30 min. N-butanol was used to extract TBA-reactive compounds; and absorbance was measured at 532 nm. The standard was MDA; and the results were given in nmol/mg protein^25^.

#### Total antioxidant capacity (TAC) assay

Samples were assessed by investigating their ability to decrease Fe^3+^ to Fe^2+^. The results were expressed in nanomoles per milligram (nmol/mg) of protein^25^. The aim of the lipid peroxidation and TAC assays was to assess the oxidative damage of Pb.

### Histological examination

#### Light microscopy (LM) examination

The aim was to assess the structural changes of the cerebrum. The cerebral samples were fixed in a 10% neutral buffered formalin solution; then tissues were processed for LM examination, and stained with hematoxylin and eosin (H&E)^26^. Five different non-overlapped sections from each animal, were examined and evaluated under LM and photographed. Ten fields from 3 different non-overlapped horizontal sections from each rat were coded enabling blind examination and evaluation. Assessment images were analyzed for the following: (1) thickness of the frontal cortex; from each section, 20 perpendicular lines between the white and pia mater were quantified at 100× magnification, (2) total number of vesicular neurons (pyramidal or granular) per field; at 400× magnification^27^.

#### Electron microscopy (EM) examination

The aim was to assess the ultrastructural changes of the cerebrum. Cerebral sections from each group were immediately placed in 3% glutaraldehyde in 0.1 M phosphate buffer for a few hours and post fixed in 1% osmium tetroxide for 1 h. Ultrathin sections of 50 nm were cut by an ultra-microtome from selected areas, were contrasted with uranyl acetate and Pb citrate^9^. Sections were examined under JEM-2100, EM unit; and images were captured using AMT CCD camera (software version AMTV600).

### Immunohistochemical analysis

GFAP immunohistochemical aimed to evaluate Pb effect on astrocytes. The streptavidin–biotin immunoperoxidase method was used for immunohistochemistry. The slides were then incubated overnight with primary the antibodies: polyclonal rabbit anti-glial fibrillary acidic protein (anti-GFAP) delivered from Sigma Laboratories was used. Universal kit used avidine biotin peroxidase system produced by Novacastra Laboratories Ltd. Incubation with a secondary antibody and product visualization were performed with diaminobenzidine chromogen. Counterstaining with Mayer’s hematoxylin and slides washing with distilled water and phosphate-buffered saline (PBS) were done. PBS was used instead of primary antibody as negative controls^28^. Ten fields from 3 different non-overlapped horizontal sections from each rat were coded enabling blind examination and evaluation under LM and photographed.

### Statistical analysis

The sample size was calculated to be 60 (10 in each group) according to Galal et al^9^. The exposed/unexposed ratio was 5/1, using open-EPI at confidence level 95% and power 80%. The SPSS 18.0 program was used to statistically analyze all the data. The Shapiro–Wilk test was used to examine if the data had a normal distribution. The mean values of various groups were compared using a one-way ANOVA, and multiple comparisons were evaluated using the Tukey HSD Post-hoc Test. Normal distributed data were reported as mean and standard deviation (SD) (Statistical Package for Social Science). Kruskal–Wallis H tests were used to compare the median values of the groups in non-normally distributed data, multiple comparisons were evaluated using the Mann–Whitney test, and non-normally distributed data were displayed as median (range). A value of *P* < 0.05 was accepted as statistically significant; and value of *P* < 0.001 was considered highly statistically significant^29^.

### Ethical approval and consent to participate

All studies and procedures involving rats were approved by Institutional Animal Care and Use Committee at Zagazig University in Egypt (ZU-IACUC/3/F/93/2020) and by the National Institute of Health (NIH) guidelines. The publication of this manuscript has been approved by all authors. We attest that we have studied the Journal's stance on matters pertaining to ethical publication and declare that our report complies with those standards.

## Results

Regarding to body weight day before study, there was no significant difference (*P*: 0.97) between all groups. In contrast, rats treated with Pb only “low-Pb and high-Pb groups” and high Pb + Se group showed a significant decrease (*P* < 0.001) in Lwt, br, and br/Lwt% in comparison to control group. Combination of Pb with Se in low Pb + Se and high Pb + Se groups showed significant increase in Lwt, br and br/Lwt% in comparison with low Pb and high Pb groups. Comparing with control group and low Pb + Se group showed no significant difference, while high Pb + Se group showed a significant decrease in Lwt, br, and br/Lwt% in comparison with other two groups (Table 1).

**Table 1:** Effect of Pb ± Se on body and brain weights in different study groups.

	Control group (n10)	Se group (n10)	Low Pb group (n10)	High Pb group (n10)	Low Pb + Se group (n10)	High Pb + Se group (n10)	*P* value
bwt (gm)	181 ± 6.24	180.5 ± 6.75	180.4 ± 8.41	180.6 ± 7.50	180 ± 6.25	182.8 ± 10	0.97
Lwt (gm)	194.3 ± 7.32	197.7 ± 8.25	178.1 ± 7.23^a^	165 ± 10.38^b^	189.6 ± 8.36^d^	181.9 ± 11.07^a,e^	< 0.001
% Of dif.^#^		0.17.5%	− 8.34%	− 15.08%	− 2.42%	− 6.38%	
br (gm)	1.79 ± 0.09	1.81 ± 0.09	1.59 ± 0.08^b^	1.46 ± 0.09^b^	1.73 ± 0.08^c^	1.64 ± 0.08^a,f^	< 0.001
% Of dif.^#^		1.12%	− 11.17%	− 18.44%	− 3.35%	− 8.38%	
br/Lwt%	0.92 ± 0.015	0.92 ± 0.009	0.89 ± 0.012^b^	0.88 ± 0.008^b^	0.91 ± 0.007^c^	0.90 ± 0.012^a,e^	< 0.001
% Of dif.^#^		0%	− 3.26%	− 4.35%	− 1.09%	− 2.17%	

One-way ANOVA, and Tukey HSD Post-hoc Test, *P* > 0.05: no significant differences, *P* < 0.05: significant differences, *P* < 0.001: highly significant differences.

^#^% of difference = mean of (each) group—mean of control group)/mean of control group %, *bwt* body weight at the beginning of the experiment, *Lwt* last day body weight**,** *br* brain weight, *br/Lwt%* brain weight/body wt. at last day%

^a^Significant vs control group, ^b^highly significant vs control group.

^c^Significant vs low Pb group, ^d^highly significant vs low Pb group.

^e^Significant vs high Pb group, ^f^highly significant vs high Pb group.

Low Pb, high Pb, low Pb + Se, and high Pb + Se groups showed a significant increase in cerebral DNA fragmentation (tailed, tail length, tail DNA and tail moment) compared to control group. Also, low Pb + Se and high Pb + Se groups showed significant decrease in cerebrum DNA fragmentation in comparison with low Pb and high Pb groups respectively (Table 2).

**Table 2:** DNA fragmentation and biochemical analysis in different study groups.

	Control group (n:10)	Se group (n:10)	Low Pb group (n:10)	High Pb group (n:10)	Low Pb + Se group (n:10)	High Pb + Se group (n:10)	*P* value
Biochemical markers							
MDA^#^ (nmol/mg)	0.6 ± 0.08	0.32 ± 0.05	3.73 ± 0.25^b^	6.14 ± 0.36^b^	1.11 ± 0.2^a,d^	4.3 ± 0.4^b,f^	< 0.001
TAC^#^ (nmol/mg)	6.7 ± 0.41	7.11 ± 0.46	2.41 ± 0.88^b^	0.39 ± 0.08^b^	5.45 ± 0.58^a,d^	2.1 ± 0.37^b,f^	< 0.001
DNA fragmentation							
Tailed (%)	2.8 ± 0.84	2.8 ± 0.84	7.4 ± 1.14^b^	11.6 ± 1.14^b^	5 ± 1^a c^	8.2 ± 0.84^b,f^	< 0.001
Untailed (%)	97.2 ± 0.84	97.2 ± 0.84	92.6 ± 1.14^b^	88.4 ± 1.14^b^	95 ± 1^a,c^	91.8 ± 0.84^b,f^	< 0.001
Tail length (μm)	1.11 ± 0.1	1.12 ± 0.02	2.29 ± 0.14^b^	3.23 ± 0.06^b^	1.3 ± 0.05^a,d^	2.5 ± 0.05^b,f^	< 0.001
Tail DNA (%)	1.13 ± 0.08	1.14 ± .03	2.17 ± 0.11^b^	3.12 ± 0.05^b^	1.3 ± 0.04^a,d^	2.3 ± 0.1^b,f^	< 0.001
Tail moment (unit)	1.21 ± 0.2	1.31 ± 0.05	5.14 ± 0.66^b^	10.09 ± 0.43^b^	2.05 ± 0.34^a,d^	5.67 ± 0.39^b,f^	< 0.001
Photos of CCD camera of comet assay							

Photos of CCD camera show effects of Pb, Se and their combinations on cerebrum comet assay in male albino rats after 4 weeks of exposure. Blue arrows = intact cells; yellow arrows = cells with a shadow, indicating damaged DNA.

^#^nmol/mg, One-way ANOVA, and Tukey HSD Post-hoc Test, P > 0.05: no significant differences, P < 0.05: significant differences, P < 0.001: highly significant differences.

^a^Significant vs control group, ^b^highly significant vs control group.

^c^Significant vs low Pb group, ^d^highly significant vs low Pb group.

^e^Significant vs high Pb group, ^f^highly significant vs high Pb group.

Regarding to biochemical analysis for MDA, low Pb, high Pb, low Pb + Se and high Pb + Se groups showed significant increase in cerebral MDA in comparison with control group. Combination of Pb with Se in low Pb + Se and high Pb + Se groups showed a significant decrease in cerebral MDA in comparison with low Pb and high Pb groups respectively. On the other hand, low Pb, high Pb, low Pb + Se, and high Pb + Se groups showed significant decrease in cerebral TAC in comparison to control group. Combination of Pb with Se in low Pb + Se and high Pb + Se groups showed a significant increase in cerebral TAC compared to the low Pb and high Pb groups, respectively (Table 3).

**Table 3:** Morphometric analysis for H&E-stained cerebral sections in different study groups.

	Control group (n:10)	Se group (n:10)	Low Pb group (n:10)	High Pb group (n:10)	Low Pb + Se group (n:10)	High Pb + Se group (n:10)	*P* value
Thickness of frontal cortex*	1033.46 ± 219.46	1052.32 ± 217.78	789.04 ± 108.66^a^	385.70 ± 97.39^b^	993.92 ± 121.36^c^	788.37 ± 111.84^a,f^	< 0.001
% Of dif.^#^		1.82%	− 23.65%	− 62.68%	− 3.83%	− 23.72%	
No. of vesicular neurons/field^&^	54.8 ± 13.85	56.3 ± 13.60	25.40 ± 6.47^b^	9.60 ± 2.43^b^	49.70 ± 12.55^d^	25.15 ± 6.78^b,e^	< 0.001
% Of dif.^#^		2.74%	− 53.65%	− 82.48%	− 9.31%	− 54.11%	

One-way ANOVA, and Tukey HSD Post-hoc Test, *P* > 0.05: no significant differences, *P* < 0.05: significant differences, *P* < 0.001: highly significant differences.

*At 100 magnification, ^&^at 400 magnification, ^#^% of difference = mean of (each) group—mean of control group)/mean of control group %.

^a^Significant vs control group, highly significant vs control group.

^c^Significant vs low Pb group, ^d^highly significant vs low Pb group.

^e^Significant vs high Pb group, ^f^highly significant vs high Pb group.

**Figure 1:**
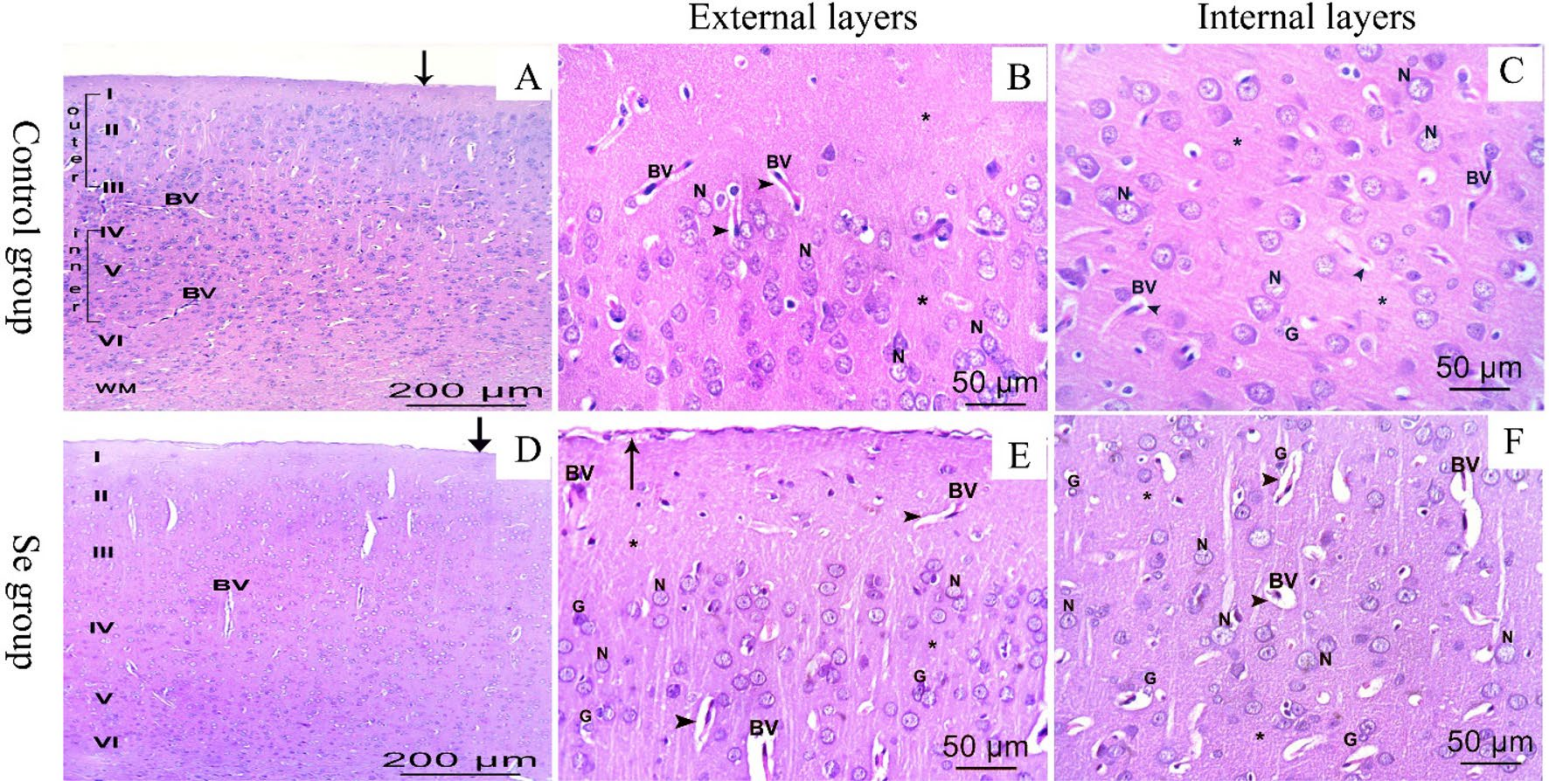
H&E photomicrographs of the cerebral tissues. Control (**A**–**C**) and Se (**D**–**F**) groups. (**A**,**D)** Layers of the cerebral cortex: (I) the molecular layer, (II) the external granular layer, (III) the external pyramidal layer, (IV) the internal granular layer, (V) the internal pyramidal layer, (VI) the multiform layer, in addition to white matter (WM), blood vessels (BV), thin and normally attached pia matter (arrow) are noticed. External layers (**B**,**E**), and internal layers (**C**,**F**) are presenting cortical neurons (N) with rounded pale nuclei and basophilic cytoplasm. Acidophilic neuropil (*) shows blood vessels (BV) with narrow perivascular spaces (arrowheads) and neuroglia cells (**G**) that are smaller with small well-demarcated nuclei (bar **A**,**D**: 200 μm ×100–**B**,**C**,**E**,**F**: 50 μm ×400).

Examination of H&E-stained cerebral sections of rats of control and Se groups showed the well-known normal histological structure of the cerebral cortex, which was arranged in six successive layers: (1) the molecular layer underneath the meninges and consisted of homogenous neuropil and few cell bodies of neurons, (2) the external granular layer, (3) the external pyramidal layer, (4) the internal granular layer, (5) the internal pyramidal layer, and (6) the multiform layer was dominated by elongated spindle shaped cells. External cortical layers both granular and pyramidal were crowded with small cortical vesicular neurons while internal cortical layers have fewer but larger neurons. Neurons had rounded pale nuclei and little basophilic cytoplasm. Glial cells appeared small with small well-demarcated nuclei. The acidophilic background (neuropil) was composed of neuronal and glial cell processes and blood vessels with a narrow perivascular space (Fig. 1A–F).

**Figure 2:**
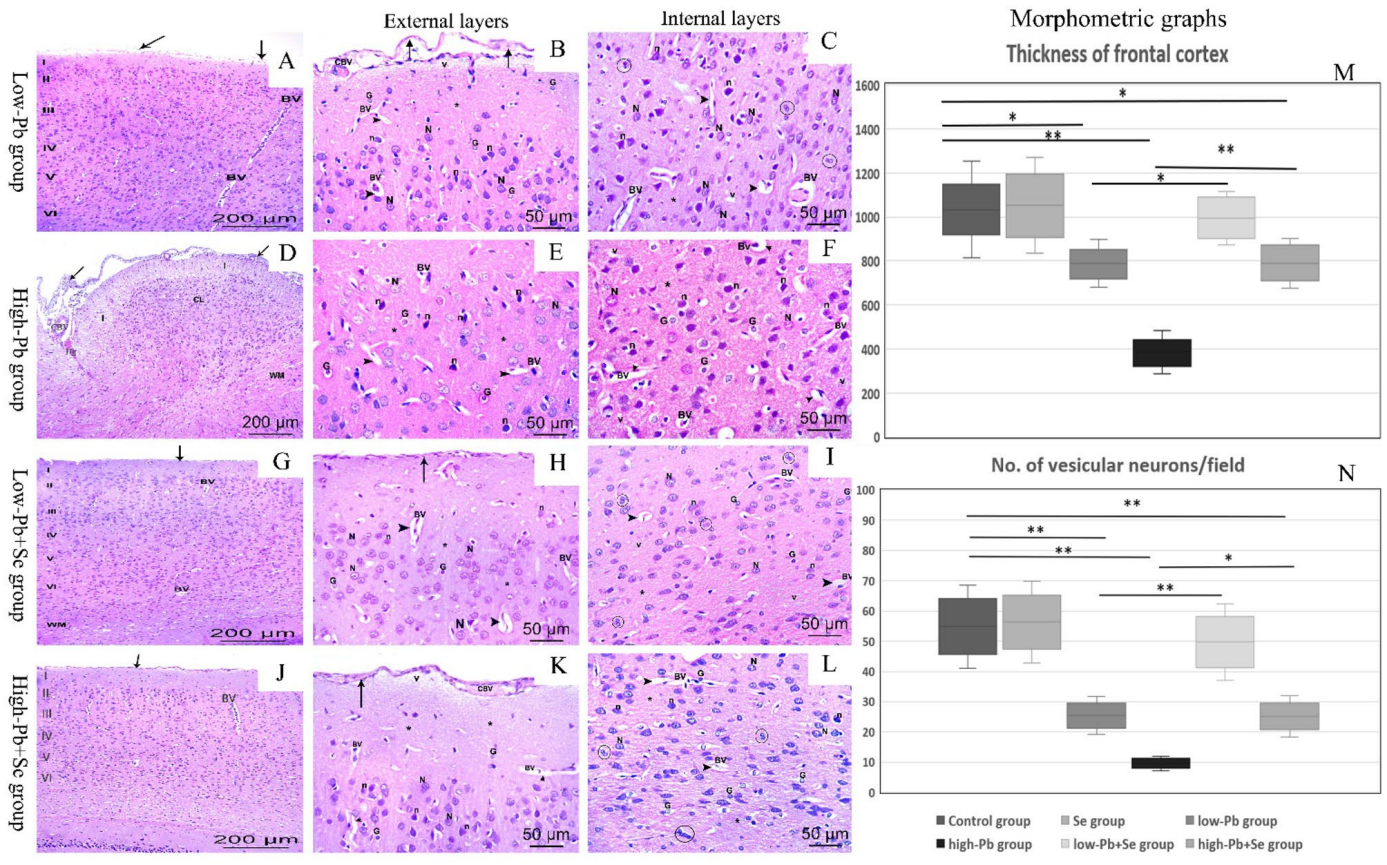
H&E photomicrographs of the cerebral tissues. Low Pb group (**A**–**C**), high Pb group (**D**–**F**), low Pb + Se group (**G**–**I**), high Pb Se group (**J**–**L**). (**A**,**G**,**J**) Micrographs show layers of the cerebral cortex I–VI. (**D)** Micrograph show severe disruption nearly in all layers (CL) with vacuolated (v) molecular layer (I). Pia matter (arrow) was irregular and separated in (**A**,**B**,**D**) and regular attached in (**G**,**H**,**J**,**K**). *WM* white matter, *Bv* blood vessels, *CBV* congested blood vessels, *arrowheads* wide perivascular spaces, *HG* hemorrhage, *N* cortical neurons with rounded pale nuclei and thin rim of basophilic cytoplasm, *n* neurons are irregular in shape with darkly stained nuclei and surrounded by pericellular halo, * acidophilic neuropil, *G* glial cells with rounded nuclei and pale areas of cytoplasm, most probably astrocytes, mitotic activity in some neuroglia (circle) (bar **A**,**D**,**G**,**J**: 200 μm ×100–**B**,**C**,**E**,**F**,**H**,**I**,**K**,**L**: 50 μm ×400). (**M**,**N)** Charts show quantitative assessment of the cortical thickness and total no. of neuron/filed. Data were expressed as mean ± SD.

Examination of H&E-stained cerebral sections of rats of low Pb group showed slight separation in pia matter with cortical layers still can be distinguished. Molecular layer was vacuolated. Most of neurons were with rounded pale nuclei and thin rim of basophilic cytoplasm while, other neurons were irregular in shape with darkly stained nuclei and surrounded by pericellular halos. Acidophilic neuropil contained blood vessels with slight wide perivascular spaces and numerous glial cells which appeared small with small well-demarcated nuclei. Moreover, some neuroglia showed sign of mitotic activity. High Pb group’s H&E-stained cerebral sections revealed marked separation of pia matter with disorganization of the six less cellular layers of the cerebral cortex and large congested blood vessels. Most of neurons were irregular in shape, darkly stained nuclei, strong acidophilic cytoplasm and surrounded by pericellular halos while, few presented large rounded pale nuclei. Acidophilic neuropil showed blood vessels with wide perivascular spaces, vacuolation, and numerous glial cells with rounded nuclei and pale cytoplasm (Fig. 2A–F).

Low Pb + Se and high Pb + Se groups’ H&E-stained cerebral sections exhibited a relatively normal appearance with regular normally attached pia matter. Neurons had rounded pale nuclei and basophilic cytoplasm while, few neurons had darkly stained irregular nuclei surrounded by pericellular halos. Acidophilic neuropil showed few vacuolation and blood vessels with wide perivascular spaces. Sign of mitotic activity in some neuroglia was observed (Fig. 2G–L).

Regarding to morphometric analysis for H&E-stained cerebral sections, low Pb, high Pb and high Pb + Se groups showed a significant decrease in frontal cortex thickness and vesicular neurons number/field in comparison to control group; while low Pb + Se group showed no significant difference with control group. Moreover, low Pb + Se and high Pb + Se groups showed significant increase in frontal cortex thickness and vesicular neurons number/field in comparison with low Pb and high Pb groups (Table 3; Fig. 2M,N).

**Figure 3:**
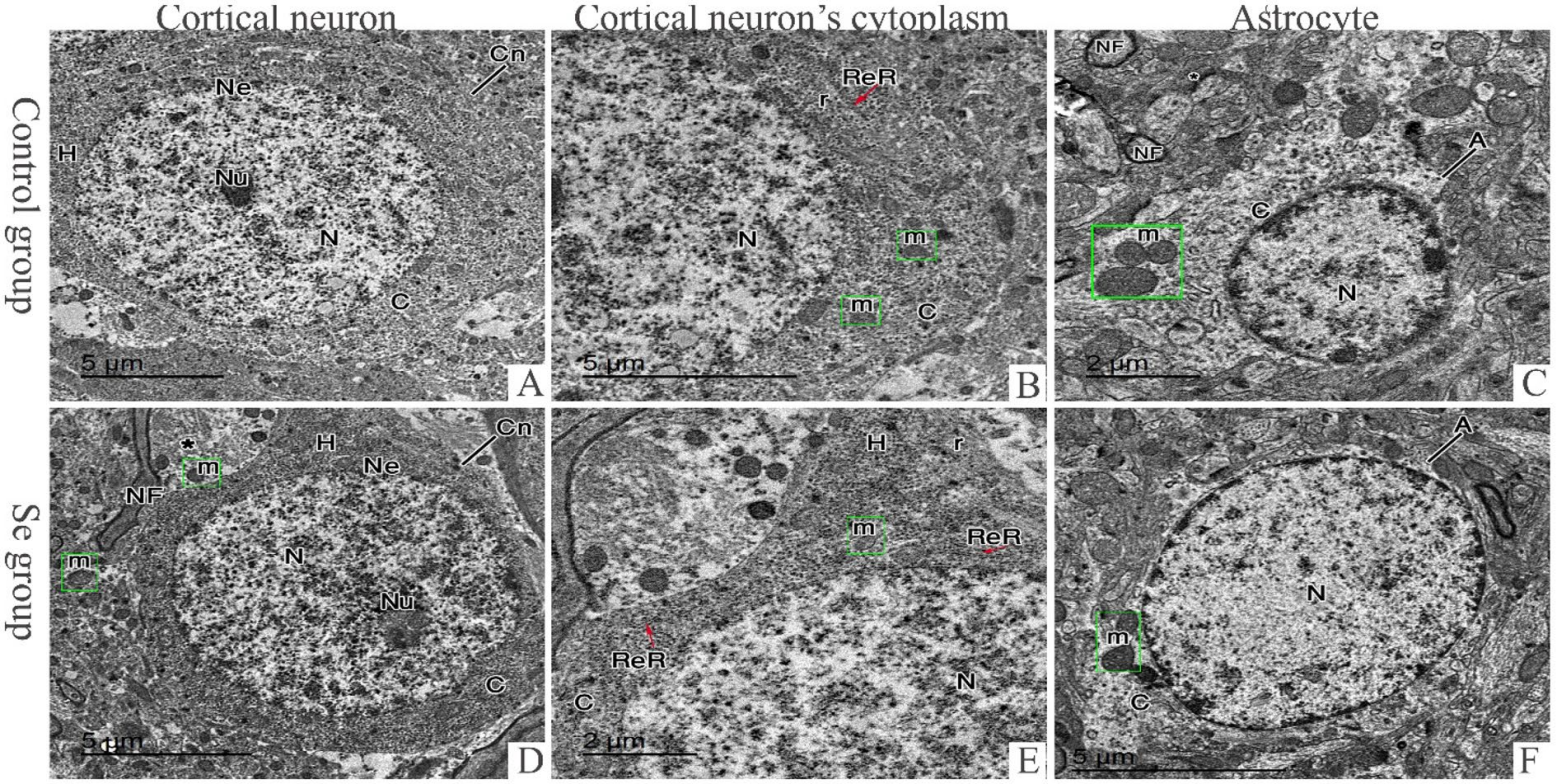
Electron photomicrographs of cerebral tissues. Control (**A**–**C**) and Se (**D**–**F**) groups. (**A**,**D)** Electron photomicrographs represent cortical neuron (Cn) with vesicular euchromatic nucleus (N) surrounded by regular nuclear envelope (Ne) and a dark prominent nucleolus (Nu). The neuropil (*) filled with regular myelinated nerve fibers (NF) and normal mitochondria (m). (**B**,**E**) Electron photomicrographs show cortical neurons’ cytoplasm (C) contains normal mitochondria (m), normal rough endoplasmic retinaculum (ReR) and free ribosomes (r). The axon hillock (H) can be observed. (**C**,**F**) Electron photomicrographs display an astrocyte (A) having a well-demarcated rounded nucleus (N) surrounded by a narrow electron lucent cytoplasm (C) that contains normal mitochondria (m). The neuropil (*) filled with regular myelinated nerve fibers (NF) (bar **A**,**B**,**D**,**F**: 5 μm ×1500 17–**C**,**E**: 2 μm ×2000 17).

**Figure 4:**
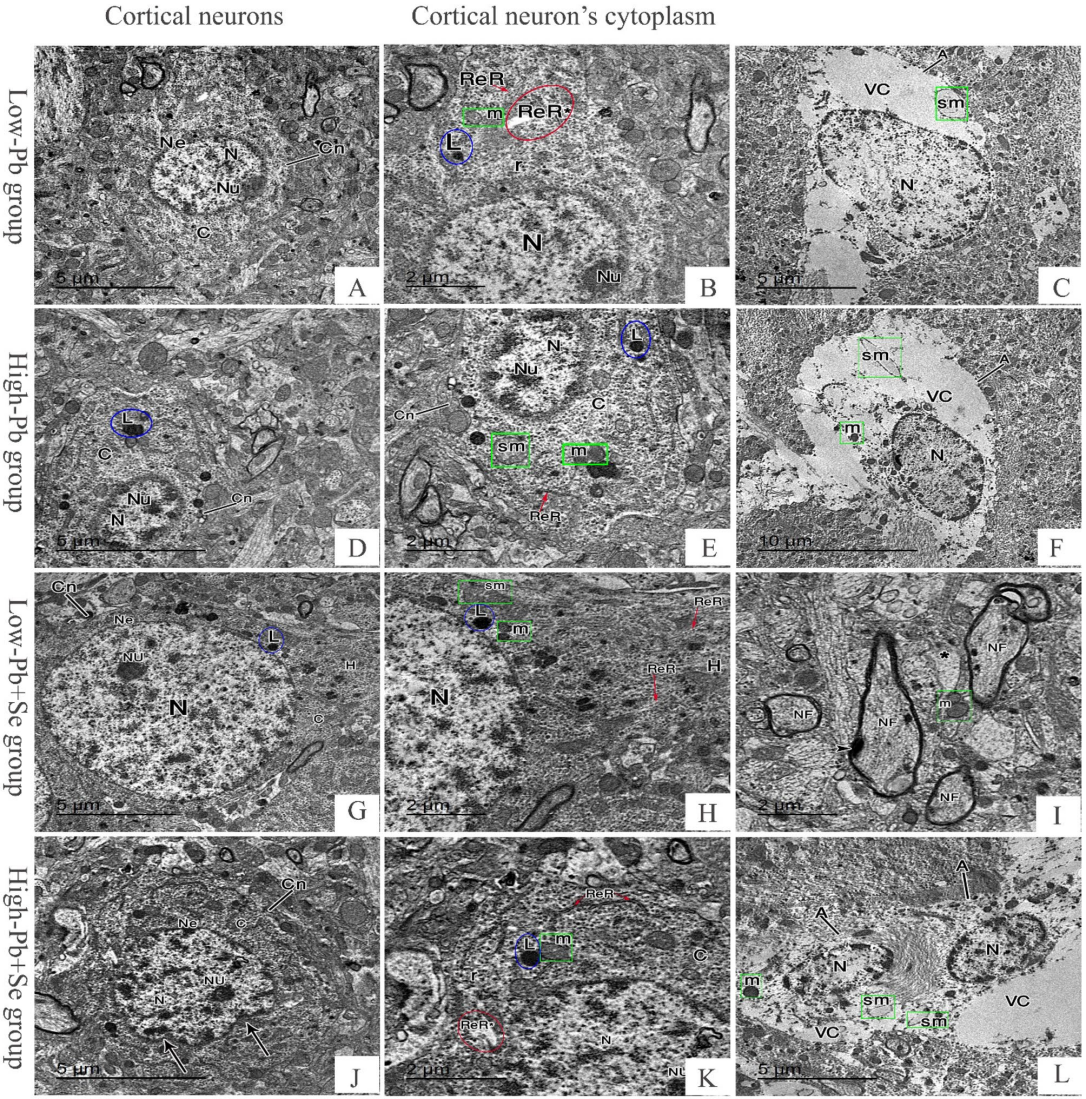
Electron photomicrographs of cerebral tissues. Low-Pb group (**A**–**C**), high-Pb group (**D**–**F**), low Pb + Se group (**G**–**I**), high Pb Se group (**J**–**L**). (**A**,**D**,**J)** Electron photomicrographs show a cortical neuron (Cn) with small shrunken heterochromatic nucleus (N) with a dark prominent nucleolus (Nu), arrows; irregularity in nuclear envelope (Ne) is irregular in (**J)**. (**G**) Electron photomicrograph show cortical neuron (Cn) with vesicular euchromatic nucleus (N) surrounded by regular nuclear envelope (Ne) and a dark prominent nucleolus (Nu), the axon hillock (H) can be observed. (**B**,**E**,**H**,**K**) Electron photomicrographs show cortical neurons’ cytoplasm. *ReR* normal rough endoplasmic retinaculum, ReR* dilated rough endoplasmic retinaculum, *m* normal mitochondria, *sm* swollen mitochondria losing its cisterna, *L* lysosomes, *r* free ribosomes. (**C**,**F**,**L**) Electron photomicrographs show astrocyte (A) with an oval well-demarcated nucleus (N) surrounded by a wide vacuolated cytoplasm (VC). (**I**) Electron photomicrograph show the neuropil (*) contains normal mitochondria (m) and myelinated nerve fibers (NF) some of them has irregularity in myeline sheath (arrowhead) (bar **F** 10 μm ×800 17 **A**,**C**,**D**,**G**,**J**,**L**: 5 μm ×1500 17–**B**,**E**,**H**,**I**,**K**: 2 μm ×2000 17).

EM examination of cerebral sections of rats of control and Se groups revealed cortical neurons with vesicular euchromatic nucleus surrounded by regular nuclear envelope with a dark prominent nucleolus. Their cytoplasm contained well developed strands of rough endoplasmic reticulum, numerous mitochondria and free ribosomes. The astrocyte cells appeared with sharply demarcated nucleus and electron lucent cytoplasm containing mitochondria. The neuropil contained regular myelinated nerve fibers (Fig. 3A–F). Cerebral sections of rats of low Pb group revealed neurons with small heterochromatic nucleus surrounded by regular nuclear envelope with a dark prominent nucleolus. Cytoplasm was dark and contained lysosomes, free ribosomes, normal mitochondria, normal rough endoplasmic retinaculum (RER), and other dilated ones. The astrocytes contained sharp demarcated nucleus with chromatin and vacuolated cytoplasm containing swollen mitochondria losing its cisterna. In high Pb group, cortical neurons were shrunken with more lysosomes and swollen mitochondria losing its cisterna (Fig. 4A–F). In low Pb + Se group, the cerebral sections showed normal cortical neurons with lysosomes and swollen mitochondria, the neuropil contained normal mitochondria and myelinated nerve fibers some of them has irregularity in myeline sheath. On the other hand, high Pb + Se group’s cerebral sections displayed cortical neurons with heterochromatic nucleus surrounded by irregular nuclear envelope and indentations (Fig. 4G–L).

**Figure 5:**
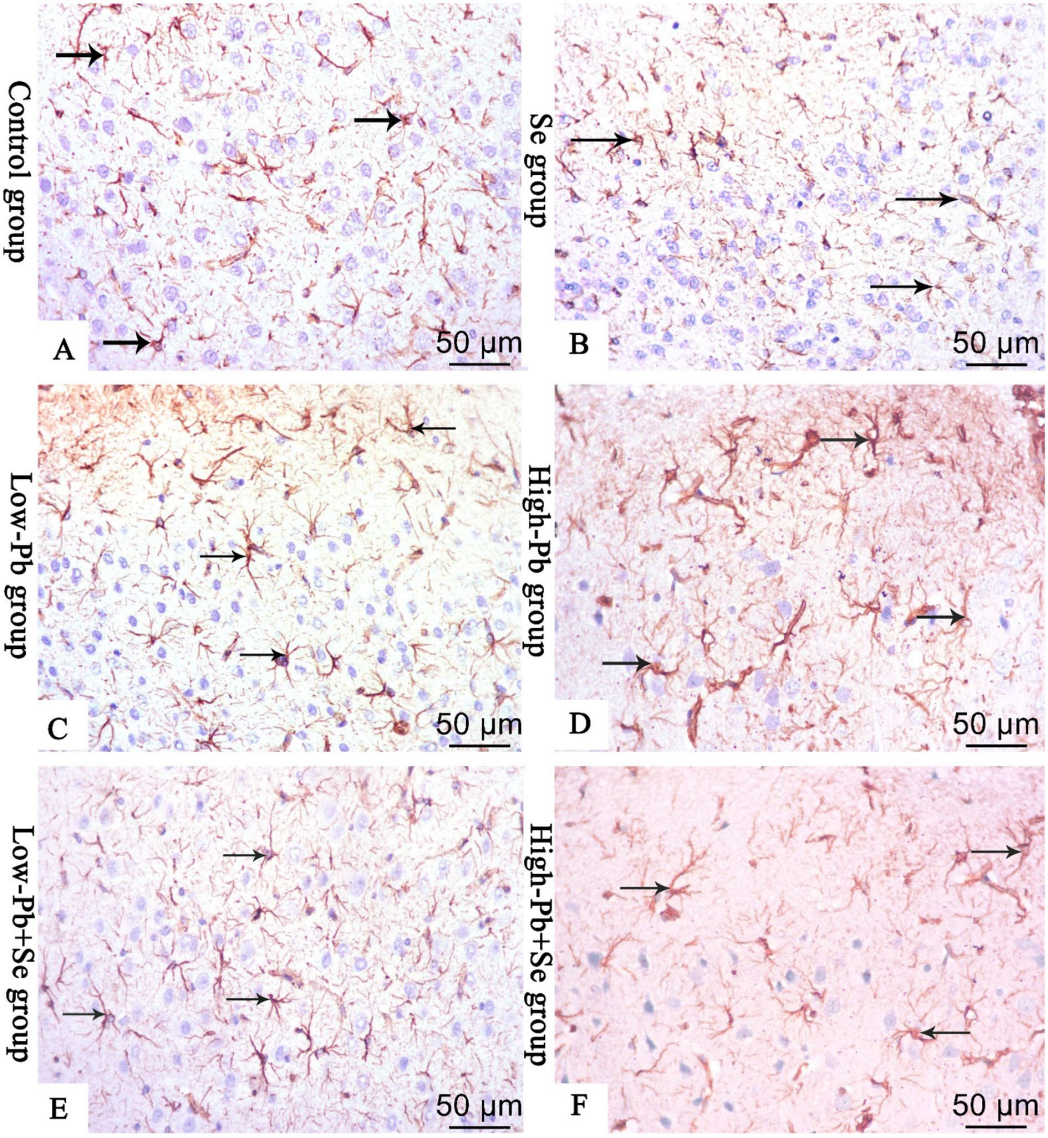
Immunohistochemical staining for anti-GFAP (**A **control group; **B **Se group; **C **low-Pb group; **D **high-Pb group; **E **low Pb + Se group; **F **high Pb + Se group). The stained cerebral sections show a positive GFAP-staining in the cytoplasm and processes of astrocytes (arrow) which appear small with thin, few short in groups (**A**) and (**B**), variable size with thick, long, branched processes in group (**C**), multiple, thick, long, branched processes in group (**D**), variable size with thin, long branched processes in group (**E**), appear with multiple, thick branched processes in group (**F**) (bar **A**–**F** 50 μm ×400).

Examination of GFAP-stained cerebral sections of rats of control and Se groups showed positive GFAP-staining in the cytoplasm of astrocytes and their processes. The cells appeared small and few with short thin few processes. On the other hand, low Pb group showed cytoplasm and processes of astrocytes appeared variable size with thick, few, and short processes. In the low Pb + Se group, the cytoplasm and processes of astrocytes appeared variable in size with thin and long branching processes. Moreover, in high Pb and high Pb + Se groups, there was much positive GFAP staining in the cytoplasm and processes of astrocytes which appeared multiple with thick, long and branched processes (Fig. 5A–F).

## Discussion

In recent years, environmental pollution with heavy metals has increased radically with the rapid development of modern industry. Excess amounts of heavy metals including Pb in animal feed and forage are often the result of human actions resulting from either agricultural or industrial production or accidental or intentional misuse^30^. The average human diet contains about 3 μg of Pb per day; 1–10% are really absorbed. In the presence of a nutritional deficiency, the degree of Pb absorption may even increase^31^. Pb poisoning is often caused by the consumption of high concentrations in drinking water^32^. Our study investigated the potential neuroprotective effects of Se against the neurotoxic effect of Pb in rats. We used male animals to avoid any potential effects of hormonal changes during estrous cycles that occur in females^33,34^.

The Pb-treated groups (low Pb group and high Pb group) displayed significant decreases in body weight, brain weight and relative brain weight compared to control and Se groups; these results are consistent with those of Singh et al.^7^. However, concomitant use of Se with Pb improved the feeding status resulting in improvement of these anthropometric measures especially in low Pb + Se group. These results contradict those of Nehru et al.^35^ who reported that Se administration has no effect on brain and body weight gain.

The brain is the organ most affected by oxidative stress due to its high rate of oxygen consumption^36^. The level of oxidative stress was measured by TAC and MDA which are end oxidation product of polyunsaturated fatty acid peroxidation. An elevated MDA level is an important indicator of lipid peroxidation and thus an indicator of oxidative stress. Oxidative stress damages the cell membrane and alters its permeability. The biochemical results of the current study showed a significant decrease in TAC and a significant increase in the level of MDA in the Pb-treated rats in both the low Pb and high Pb groups. These results are in general agreement with other studies^7,10,37^. Our biochemical data reinforced that Se is a potent antioxidant as there was significant decrease in MDA level and significant increase in TAC level in rats of Pb + Se treated groups (low Pb + Se and high Pb + Se groups) compared to the Pb-only groups. These results are generally consistent with other studies investigating the role of Se in the alleviation of oxidative stress induced by chromium (VI), cypermethrin, and 1-methyl-4-phenyl-1,2,3,6-tetrahydropyridine (MPTP) ^13,38,39^. This antioxidant effect of Se is proved by Rahbardar et al.^40^, who confirmed increased glutathione content, and reduced MDA level. Moreover, these results agree with Zakeri et al.^6^ who stated that Se has antioxidant, anti-inflammatory, and antiviral properties, and with Shahidi et al.^41^ who showed that administration of Se to rats with Alzheimer disease led to an increase in the hippocampal TAC, a decrease in MDA level and an increase in recovered memory with improved cognition. These results are not consistent with those of Gholamigeravand et al.^42^ who reported that Se administration did not significantly increase TAC, but reduced MDA.

Separation of pia matter was detected in rats in low Pb and high Pb groups. This was demonstrated by AL-Mzaien et al.^30^ who reported that this segregation may contribute to edema as a result of oxidative stress resulting to a change in permeability leading to abnormalities in hemodynamics and fluid leakage into neural tissue. Some authors suggested that BBB disturbance is a causative mechanism in Pb neurotoxicity^43–45^. In case of co-administration of Se with Pb, the pia matter was found to be regular and normally attached in low Pb + Se group but still slightly detached in high Pb + Se group.

Oxidative stress regulates apoptosis associated with the activation of the intrinsic pathway of apoptosis within the mitochondria. Polyunsaturated fatty acids in plasma and organelles are the target of oxidative agents. Oxidative DNA damage induced by ROS leads to cell injury^46^. In our study, Pb has a toxic effect on the cerebral cortex of rats by inducing apoptosis which has been demonstrated histologically and biochemically. Histological examination of brain sections stained with H&E showed a change in normal cerebral architecture in the low Pb and high Pb groups. Most of the cortical neurons were shrunken with dark stained nuclei and surrounded by a peripheral halo. The vacuolar halo may be attributed to cell shrinkage and regression of cell processes as a result of the strain of the cytoskeleton creating pericellular spaces. Moreover, reduction in the total number of cortical neurons as well as thickness of frontal cortex morphometrically confirmed apoptosis. EM examination of rats’ cerebral sections of Pb treated groups revealed small shrunken cortical neurons with heterochromatic nucleus. This finding is in harmony with Galal et al.^9^. The degenerative changes detected in the neurons as well as nuclear changes in the form of shrinking and darkening (pyknosis) seen in our study reflect a certain stage of apoptosis; this result is in accordance with Afifi and Embaby^47^. In addition, ultrastructural examination of cerebral sections of low and high Pb groups revealed that the cytoplasm of cortical neurons contained many lysosomes, swollen mitochondria losing their cisterna, normal RER and another dilated RER. The astrocytes contained sharp demarcated nucleus with chromatin and vacuolated cytoplasm containing swollen mitochondria losing cisterna. Wakabayashi^48^ documented that swollen mitochondria with partial or complete loss of cristae might be correlated to oxidative stress. The detected mitochondrial changes may be measured as an early manifestation of apoptosis and an adaptive progression to undesirable surroundings due to extra exposure of the cell to free radicals at the level of intracellular organelles^49^.

Biochemically, our study showed a significant increase in apoptosis index “DNA fragmentation”; this result agrees with Khalaf et al.^50^ and Shaban et al.^51^. This Pb-apoptotic effect was approved by Zhou et al.^52^ who reported that Pb increases Bcl-2 associated X protein (Bax) expression and the ratio of Bax to B-cell lymphoma-2 (Bcl-2) “Bax/Bcl-2”.

The co-administration of the Se with the Pb produced different degrees of improvement in the histopathological changes of H&E-stained sections of rat's cerebral cortex, and protected cerebrum from Pb-apoptotic effect. This was signified by improvement in neuronal status in several areas with moderate restoration of the normal cerebral cortex organization layers. These results are in harmony with others^14,53,54^. This was confirmed by increased number of vesicular neurons number neurons and thickness of frontal cortex morphometrically. Also, EM examination of cerebral sections of rats of Pb and Se treated groups (low Pb + Se and high Pb + Se groups) revealed improvement in cortical neurons status. Cortical neurons with heterochromatic nucleus and a dark prominent nucleolus. The nuclear envelope was regular in (low Pb + Se group) and irregular showing indentations in (high Pb + Se group). Their cytoplasm was dark containing lysosomes, normal mitochondria, free ribosomes, normal RER, and little dilated RER. The Se-protective effect is confirmed by a significant decrease in DNA fragmentation percentage in groups treated with Pb and Se (low Pb + Se and high Pb + Se groups). Our results in DNA fragmentation are in synchronization with those of Khalaf et al.^55^ and Sadek et al.^56^. Other authors have said that Elsie is a double-edged sword with both beneficial and toxic effects^57,58^. Despite its importance in cell functions and as an antioxidant, excess intake can be fatal, leading to a serious disease called selenosis. Moreover, excessive exposure to Se even at low levels may lead to adverse effects on human health^58^.

Astrocytes are a target of Pb toxicity^59^. GFAP is an intermediate filament protein which is expressed by several cell types of CNS including astrocyte cells. GFAP is involved in some significant CNS functions, like cell communication and the effectiveness of the BBB. Also, GFAP has been shown to play an important role in mitosis by regulating the filament network present inside the cells^60^. Sections stained with GFAP immunohistochemistry of the Pb-treated groups showed rich positive GFAP staining of cytoplasm and astrocyte processes. The cells were increased in number and appeared larger with multiple thick processes in comparison to control and Se groups. GFAP is an indicator protein for astrogliosis. Astrocytes make up the majority of the neuroglial cells, which respond rapidly to many neurodegenerative changes, resulting in marked astrogliosis^61^. This result agrees with other studies reporting that the increase in GFAP-immunopositive astrocytes is a compensatory mechanism against neuronal cell damage^62,63^. In contrast to our study, Cai et al.^58^ reported that the GFAP-immuno-positive astrocyte numbers were significantly decreased after Pb acetate exposure. The cells appeared small with few short thin processes in control and Se groups compared to Pb-treated groups. This agrees with the study of Ibrahim et al.^64^ who stated that the GFAP reaction was significantly increased in the cyclophosphamide group and significantly decreased when combined with nano-Se.

Study limitations include using only a single dose of Se administered as 0.25 mg/kg/day to examine its protective role against cerebral Pb poisoning. However, our main goal was to investigate its potential efficacy against neurotoxicity of both low and high doses of Pb exposure through various methods including biochemical and immunohistochemical and histological screening. Although Se is essential to life, its safety is limited with regard to its dosage levels. In other words, toxic dose levels are close to those normally required for the body's needs or appropriate treatment^11^. Therefore, determining its dosage is crucial. Future studies to investigate different doses of Se in this regard are recommended.

In conclusion, the oxidative stress and apoptosis pathways have important roles in Pb-neurotoxicity. The Se is suggested to significantly improve Pb-induced histopathological changes by modulating oxidative stress and apoptosis. However, it is recommended for use in humans after more prospective experimental studies are conducted to evaluate the most effective doses that could be taken as prophylaxis or treatment for lead neurotoxicity.

## Data Availability

The data and datasets used and/or analyzed during the current study are available from the corresponding author upon reasonable request.
